# Deleterious Effects of High Dose Connexin 43 Mimetic Peptide Infusion After Cerebral Ischaemia in Near-Term Fetal Sheep

**DOI:** 10.3390/ijms13056303

**Published:** 2012-05-22

**Authors:** Joanne O. Davidson, Colin R. Green, Louise F. B. Nicholson, Laura Bennet, Alistair J. Gunn

**Affiliations:** 1 Department of Physiology, The University of Auckland, Auckland 1023, New Zealand; E-Mails: joanne.davidson@auckland.ac.nz (J.O.D.); l.bennet@auckland.ac.nz (L.B.); 2 Department of Ophthalmology, The University of Auckland, Auckland 1023, New Zealand; E-Mail: c.green@auckland.ac.nz; 3 Department of Anatomy, The University of Auckland, Auckland 1023, New Zealand; E-Mail: lfb.nicholson@auckland.ac.nz

**Keywords:** fetus, ischaemia, hemichannels, gap junctions, connexins, mimetic peptide

## Abstract

Hypoxic-ischaemic brain injury at birth is associated with 1–3/1000 cases of moderate to severe encephalopathy. Previously, we have shown that connexin 43 hemichannel blockade, with a specific mimetic peptide, reduced the occurrence of seizures, improved recovery of EEG power and sleep state cycling, and improved cell survival following global cerebral ischaemia. In the present study, we examined the dose response for intracerebroventricular mimetic peptide infusion (50 μmol/kg/h for 1 h, followed by 50 μmol/kg/24 h (low dose) or 50 μmol/kg/h for 25 h (high dose) or vehicle only (control group), starting 90 min after the end of ischaemia), following global cerebral ischaemia, induced by 30 min bilateral carotid artery occlusion, in near-term fetal sheep (128 ± 1 days gestation). Both peptide infusion groups were associated with a transient significant increase in EEG power between 2–12 h after ischaemia. The ischaemia-low dose group showed a significant recovery of EEG power from day five compared to the ischaemia-vehicle and -high dose groups. In contrast, the high dose infusion was associated with greater secondary increase in impedance (brain cell swelling), as well as a trend towards a greater increase in lactate concentration and mortality. These data suggest that higher doses of connexin mimetic peptide are not beneficial and may be associated with adverse outcomes, most likely attributable to uncoupling of connexin 43 gap junctions leading to dysfunction of the astrocytic syncytium.

## 1. Introduction

Hypoxia-ischaemia around the time of birth is associated with approximately 1–3/1000 cases of moderate to severe encephalopathy and a high risk of death or disability [1,2]. Hypoxia-ischaemia is characterized by progressive evolution of damage over days following the insult [3,4]. The mechanisms underlying this striking spread of injury are not understood, although gap junctions are believed to contribute through a “gap junction mediated bystander effect” [5,6]. Gap junctions are intercellular channels that link the cytoplasm of adjacent cells, permitting the exchange of small molecules and ions. Non-specific, global gap junction blockers such as carbenoxolone, glycyrrhizic acid, heptanol and octanol are reported to provide neuroprotection *in vitro* [7,8], after stroke in adult rats [9] and in rat pups after intrauterine hypoxia-ischaemia [10]. However, this remains controversial with connexin 43 knockout mice showing greater injury following ischaemia compared to control animals [11]. In the brain, connexin 43 (Cx43) is the predominant astrocytic connexin and has been shown to be up-regulated in human post-mortem tissue and in near-term fetal sheep after cerebral ischaemia [12,13].

Evidence now implicates astrocytic connexin hemichannels in the propagation of injury. Hemichannels, or connexons, are half of the gap junction channel that sits in the unopposed membrane of a cell, prior to the formation of new channels. They are believed to remain closed under normal physiological conditions but they may open following oxygen glucose deprivation, metabolic inhibition or low extracellular calcium ion (Ca^2+^) concentrations [14–18]. Hemichannel opening has been associated with disruption of the resting membrane potential, release of cytotoxic levels of ATP [19] and glutamate [20], and uptake of water resulting in cell swelling and rupture [21,22]. ATP has been implicated in neuropathic pain and inflammation [23], and in spinal cord injuries its release in peritraumatic areas causes excessive neuronal firing, followed by irreversible increases in intracellular Ca^2+^ and cell death [24]. In cortical astrocytes these events are specifically Cx43 hemichannel related and can be blocked with La^3+^ or peptides that block Cx43 hemichannels; treatments that block pannexin channels have no effect [18].

We recently reported that prolonged blockade of connexin 43 hemichannels with a specific mimetic peptide (50 μmol/kg/h for 1 h, followed by 50 μmol/kg/24 h), significantly improved neurological outcomes in the near-term fetal sheep following 30 min of cerebral ischaemia [13]. This included a reduction in seizures and the incidence of *status epilepticus*, improved recovery of electroencephalogram (EEG) power and sleep state cycling and improved survival of oligodendrocytes with intermediate neuronal survival between sham controls and vehicle treated animals [13]. In contrast, infusion of a mimetic peptide of the same size that does not block hemichannels *in vitro* had no significant effect compared to ischaemia-vehicle infusion. The turnover of CSF is much faster in the near-term fetus than in adults [25], and permeability of the CSF-brain barrier is maximal in the first few hours immediately after ischaemia, and then decreases [26]. We therefore hypothesised that uptake of peptide would be reduced over time after ischaemia, and that a relatively large ongoing rate of peptide delivery would be needed to maintain stable ECF levels in the brain during the secondary phase of brain injury.

Thus, in the present study we aimed to evaluate a log-order dose response curve for connexin mimetic peptides in the chronically instrumented near-term fetal sheep at 0.85 gestation. Brain maturation at this age in the sheep is equivalent to the human infant at term [27,28]. This well characterized paradigm allows long-term, real-time monitoring of many physiological parameters including brain activity and temperature, carotid artery blood flow, heart rate, blood pressure and body movements [4,29]. The primary outcome of the present study was recovery of EEG power. Clinically, there is considerable evidence that greater recovery of EEG activity after perinatal hypoxia-ischaemia is associated with better neurodevelopmental recovery [30,31], and in the fetal sheep that EEG power is correlated with neuronal survival [4,13].

## 2. Results and Discussion

### 2.1. Biochemistry and Survival

There were no significant differences in baseline blood gas data, pH, glucose and lactate between groups (Table 1). Glucose concentrations were significantly elevated from baseline in the ischaemia-vehicle and -low dose groups between two hours and one day after ischaemia (*p* < 0.05). In the ischaemia-high dose group glucose levels were only significantly elevated from baseline at day one (*p* < 0.05). There was a significant increase in lactate concentrations after ischaemia in all groups at day one (*p* < 0.05), which was not significantly different between groups. A significant reduction in PaO_2_ was seen in the ischaemia-high dose group at day one (*p* < 0.05).

All animals in the ischaemia-vehicle and -low dose groups survived the occlusion and recovery periods (7/7 and 6/6 respectively). In the ischaemia-high dose group all animals survived the occlusion period but two animals died between days two and three after ischaemia (2/7). Both of the animals that died had very high plasma lactate concentrations at day one (18.5 and 20.8 mmol/L) and at day two (8.11 and 11.9 mmol/L) before death. A third fetus developed an elevated lactate level at day one (11.2 mmol/L) but this resolved by day two (0.9 mmol/L).

### 2.2. Brain Activity and Impedance

**Figure 1:**
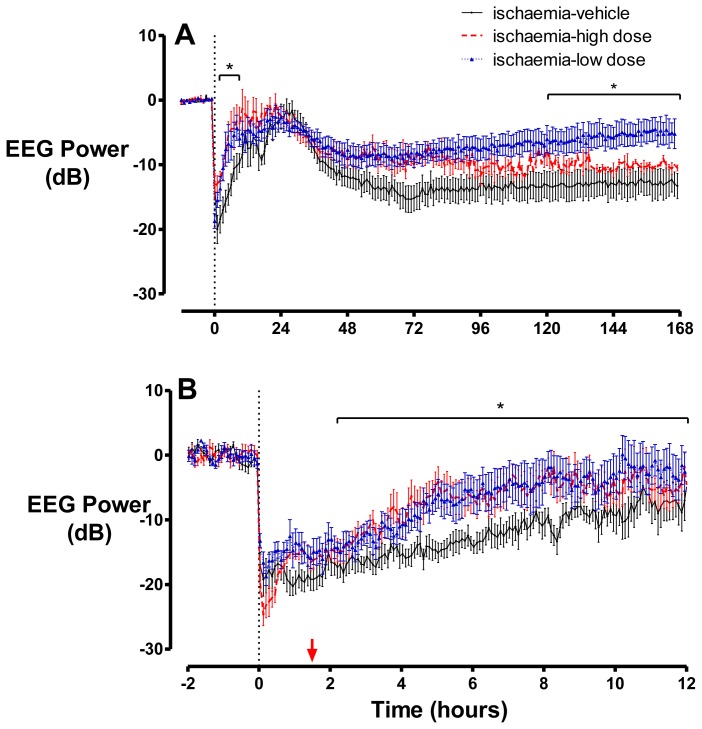
EEG power shown as hourly averages from 12 h before until seven days after ischaemia (**A**), and five minute averages from two hours before until 12 h after ischaemia (**B**), in the ischaemia-vehicle, -low dose and -high dose groups, where peptide infusion was started 90 min after the end of ischaemia (the start of infusions is shown by the red arrow). Following the onset of occlusion, EEG power is rapidly suppressed in all three groups. A secondary rise in EEG power was seen in all groups, although this was significantly greater between 2–12 h in the ischaemia-low and -high dose groups. This was followed by a reduction in EEG power to below baseline levels, which persisted at day seven. Data are mean ± SEM. * *p* < 0.05.

There were no significant differences in absolute baseline EEG power between groups (ischaemia-vehicle 22.3 ± 1.3 *vs.* ischaemia-low dose 19.8 ± 3.1 *vs.* ischaemia-high dose 20.9 ± 1.1 dB). Cerebral ischaemia was associated with rapid, profound suppression of EEG power in all groups (Figure 1), with no significant difference in EEG power during ischaemia. After release of occlusion, EEG activity gradually increased from its nadir, but remained suppressed compared to baseline for approximately eight hours in the ischaemia-vehicle group and then increased simultaneously with the onset of a period of intense seizure activity that lasted until approximately 48 h after ischaemia. In both of the groups that received peptide, EEG power was significantly higher between 2–12 h after ischaemia compared to the ischaemia-vehicle group (*p* < 0.05). There was no significant difference in the time of seizure onset between the ischaemia-vehicle (285 ± 63 min), ischaemia-low dose (258 ± 32 min) and ischaemia-high dose (373 ± 63 min) groups (*p* > 0.05). There was a weak inverse correlation between the average amplitude or total duration of seizures, during the peak period of 22–26 h, with a peak lactate concentration at day one in the ischaemia-high dose group (*r*^2^ = 0.37 and *r*^2^ = 0.39, respectively). Following the resolution of seizures, total EEG power fell to below baseline levels in all groups. However, in the ischaemia-low dose group EEG power returned towards baseline and was significantly higher than the ischaemia-vehicle and-high dose groups from day five until the end of the experiment.

**Figure 2:**
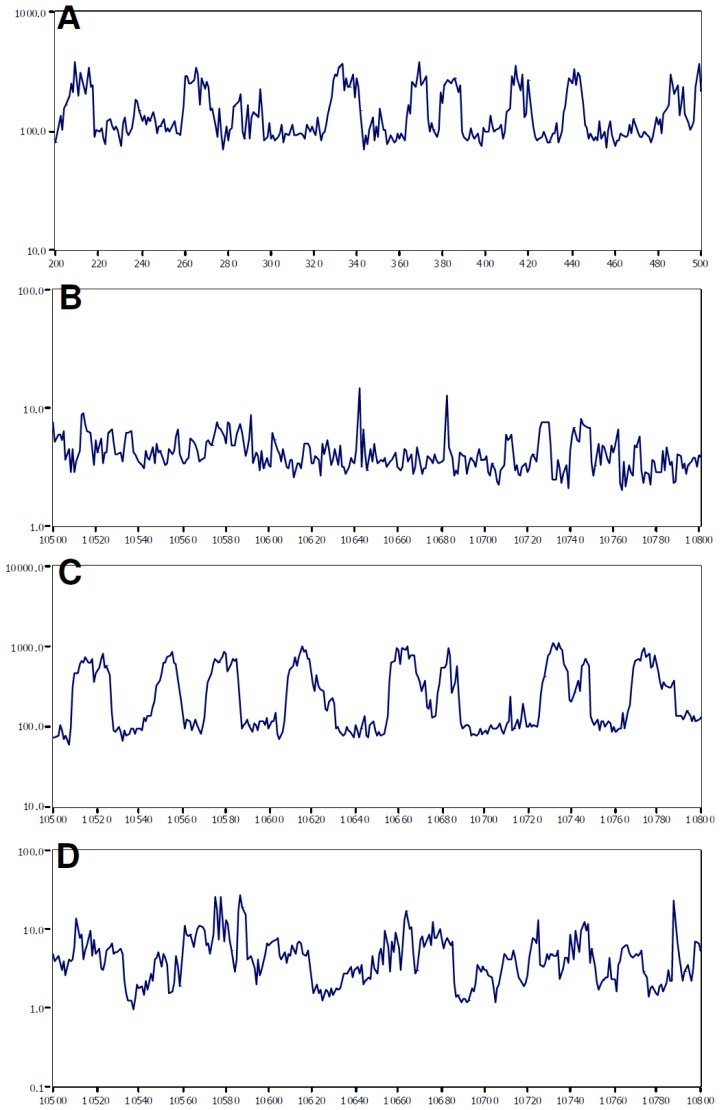
Examples of one minute averaged EEG data from individual fetal sheep showing (**A**) the typical pattern of alternating periods of low and high amplitude EEG activity characteristic of sleep state cycling during the baseline period. (**B**–**D**) One minute averaged EEG data on day seven in the ischaemia-vehicle (**B**), ischaemia-low dose (**C**) and ischaemia-high dose (**D**) groups, with clear sleep state cycling only being seen in the ischaemia-low dose group.

The normal near-term fetal EEG characteristically shows sleep state cycling (Figure 2A). In the ischaemia-vehicle and-high dose groups EEG power remained reduced and no sleep state cycling was evident at day seven (Figure 2B, D). In contrast, in the ischaemia-low dose group EEG power was near baseline values and clear sleep state cycling was present (Figure 2C).

**Figure 3:**
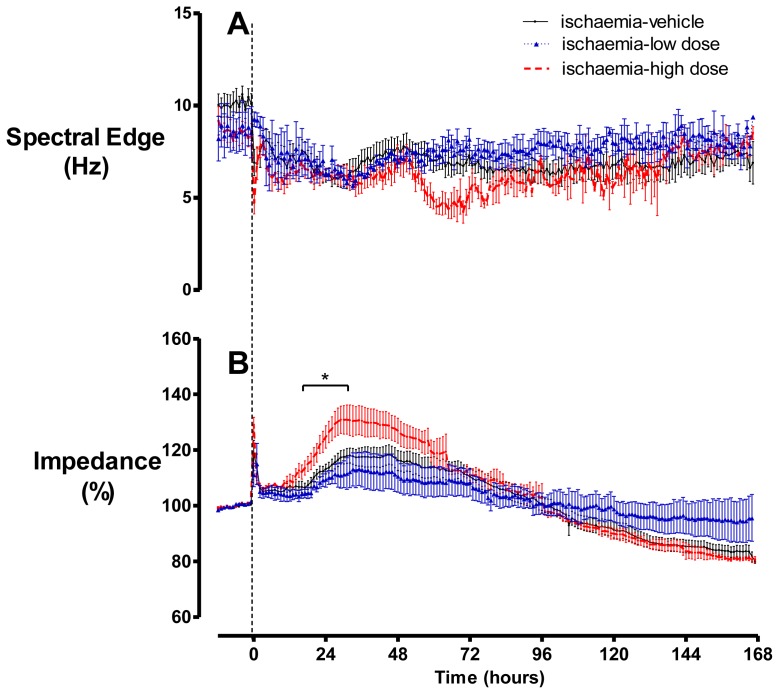
(**A**) Spectral edge was suppressed at the onset of occlusion and remained below baseline in all groups. (**B**) Impedance increased during occlusion in all groups. After release of occlusion, impedance returned towards baseline, followed by a secondary increase that was significantly greater in the ischaemia-high dose group. Data are mean ± SEM, *p* < 0.05.

There were no significant differences in baseline EEG spectral edge between groups (Figure 3). Ischaemia was associated with rapid suppression of spectral edge to below baseline levels, which persisted to day seven in all groups. An apparent transient decrease in spectral edge was seen between 48–72 h after occlusion in the ischaemia-high dose group, which was associated with data dropout due to the death of two fetuses.

Cerebral impedance (a measure of intracellular oedema [32]) increased progressively from approximately five minutes after the start of occlusion (Figure 3). There was no significant difference between the maximum impedance during ischaemia in the ischaemia-vehicle (152.8 ± 3.7%), ischaemia-low dose (145.3 ± 5.5%) and ischaemia-high dose (154.8 ± 3.4%) groups (*p* > 0.05). After release of occlusion, impedance resolved to near baseline levels over 30 to 45 min. From approximately 18 h onwards a secondary rise in impedance developed in all groups, to a significantly greater peak in the ischaemia-high dose group than the other groups (*p* < 0.05). By day seven, impedance had dropped below baseline in the ischaemia-vehicle and -high dose groups, whereas it appeared to remain at approximately baseline values in the ischaemia-low dose group (N.S.).

### 2.3. Cardiovascular Parameters, Body Movements and Temperature

**Figure 4:**
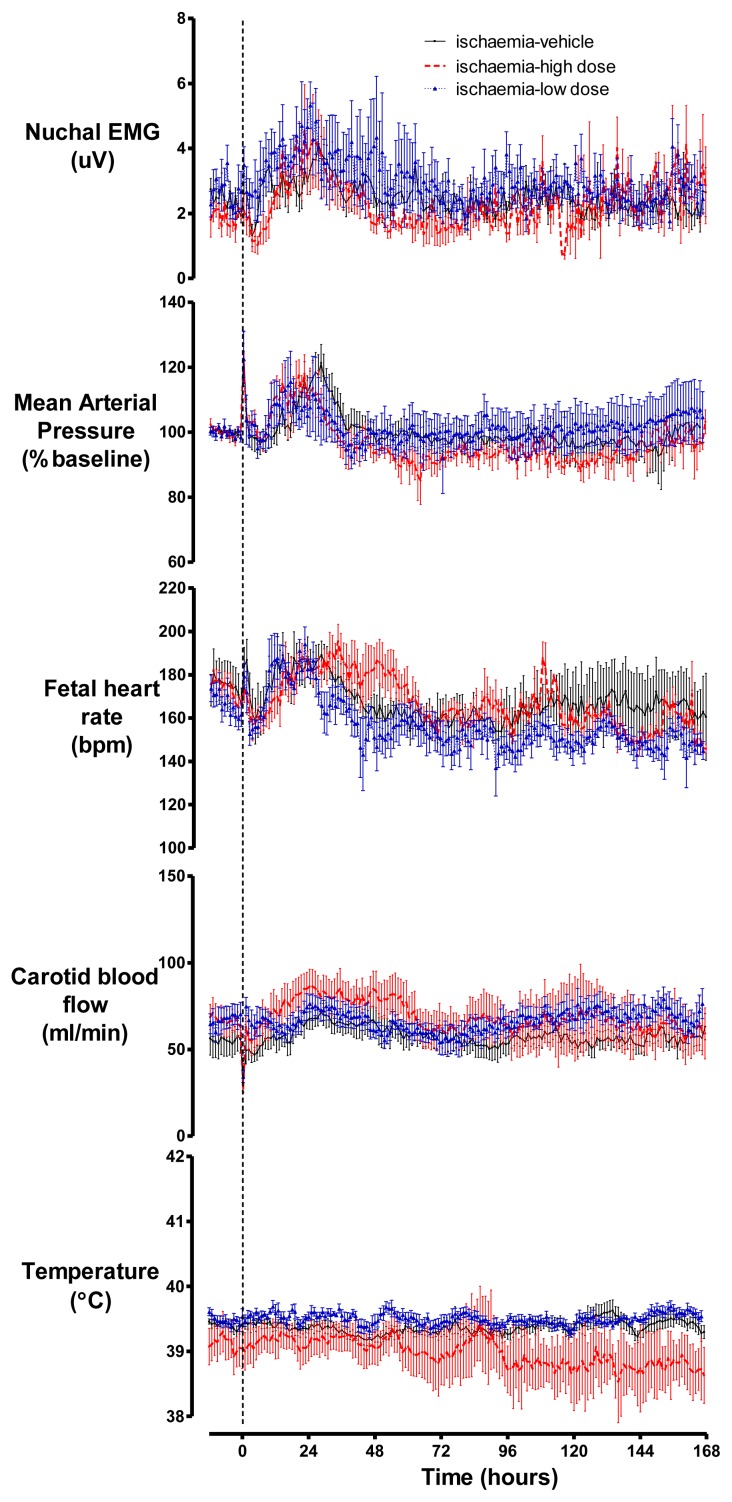
There were no significant differences in nuchal EMG, mean arterial pressure, fetal heart rate, carotid artery blood flow or brain temperature between the ischaemia-vehicle, -low dose and-high dose groups. Data are mean ± SEM.

In all groups, carotid artery occlusion was associated with a significant fall in carotid artery blood flow and an increase in fetal heart rate and mean arterial pressure compared to baseline (*p* < 0.05, Figure 4). Following the release of the occlusion these parameters returned to baseline values. A secondary rise in fetal heart rate, mean arterial pressure, nuchal electromyogram (EMG) and carotid blood flow was seen at approximately 18–36 h. There was no significant difference in any parameter between the groups. Extradural temperature was approximately 39.5 °C, with a small diurnal fluctuation, with no significant difference between groups.

### 2.4. Discussion

Consistent with our previous report, a low-dose dose mimetic peptide infusion started 90 min after reperfusion from severe cerebral ischaemia was associated with markedly improved recovery of EEG power [13]. In contrast, high-dose infusion of this peptide was not associated with any apparent improvement in EEG recovery, but instead with a significantly greater secondary rise in cerebral impedance (brain swelling), lower blood oxygen levels, and apparent trends to increased plasma lactate values and fetal death. Bilateral carotid artery occlusion is an exclusively cerebral insult, and although it is associated with severe neural injury, there is no systemic compromise [4,32].

There is increasing evidence to suggest that astrocytes play an important role in synaptic function. Astrocytes respond to the synaptic release of glutamate with an increase in intracellular Ca^2+^ and can also modulate neuronal excitability via the release of neuroactive substances called gliotransmitters [33–35]. These gliotransmitters include glutamate, ATP, D-serine and tumour necrosis factor α [36–39]. Such research has led to the concept of the “tripartite synapse”, consisting of astrocytes along with the pre- and post-synaptic terminals [33]. This has been shown to occur when an astrocyte releases glutamate that acts on both the pre- and post-synaptic terminals to modulate synaptic transmission and excitability at excitatory and inhibitory synapses [40–43]. Astrocytic ATP release can modulate excitability via purinergic receptors, whilst ATP that is degraded to adenosine can mediate depression of excitatory synapses [44,45]. It is possible that the early increase in EEG power seen in both the low- and high-dose peptide infusion groups was a result of reduced ATP release from astrocytes via connexin hemichannels. Intriguingly, this early increase in EEG power does not seem to have affected the longer-term recovery of EEG power, which was significantly improved after low-dose but not high-dose infusion. This is consistent with reports both in human infants and in large animals that EEG amplitude during the immediate recovery phase has relatively limited predictive value [46,47].

The relationship between hemichannel and gap junctional activity likely varies with the precise environmental conditions. In cell culture, hypoxia enhanced connexin hemichannel opening but reduced gap junction communication [18]. Conversely, Cotrina and colleagues have demonstrated that gap junction communication remains intact during ischaemia in brain slices [37,48]. Physiological increases in Ca^2+^ have been shown to inhibit gap junction permeability in HeLa cells [49]. Although intracellular Ca^2+^ levels increase rapidly during ischaemia [50], which could reduce gap junction coupling [49], after reperfusion, intracellular Ca^2+^ concentration rapidly falls to baseline level, and is therefore unlikely to affect gap junction coupling in the latent phase.

The mechanism of loss of protection with high-dose infusion of connexin mimetic peptide is unknown, but it is highly likely to be related to blockade of the connexin 43 gap junctions in addition to connexin hemichannels. This is supported by evidence from O’Carroll *et al.*, who showed loss of dye transfer between cells, when the same connexin mimetic peptide as used in this study was applied at 500 μM but not 5 μM doses [51]. In addition to the dose response, there may also be a time course response for mimetic peptide blockade of connexin hemichannels and gap junctions. Gap26, a mimetic peptide which binds to the first extracellular loop of connexin 43, has been shown to block connexin 43 hemichannel currents in less than five minutes in single cells and then gap junction currents after approximately 30 min in cell pairs, following application at a relatively high dose (175 μM) [52,53]. However, that study did not test whether this sequence also occurred at lower doses. Indeed, low-dose Gap26 (0.5 μM) applied either 10 min before or 30 min after the initiation of 40 min of myocardial ischaemia was shown to reduce myocardial infarct size by 48% and 55%, respectively, suggesting hemichannel blockade after ischaemia might be of relatively greater importance than during ischaemia [54,55].

Although transient blockade of gap junctions following ischaemia or injury may be beneficial [9,10,56], long-term blockade, such as that seen in the connexin 43 knockout mouse, has been associated with greater brain injury following ischaemia [11]. Thus, blockade of connexin 43 gap junctions may compromise the astrocytic syncytium and impair vital functions. These functions include glutamate uptake from the extracellular space via the transporters GLT-1 and GLAST, and conversion of glutamate to glutamine which is then released for re-uptake by neurons [57]. Astrocytes are also responsible for spatial buffering of K^+^, whereby uptake of K^+^, cytosolic diffusion through the astrocytic syncytium, followed by release at a distant site, prevents ionic imbalances in active regions [58]. Extracellular K^+^ under normal conditions is approximately 2.7–3.5 mM but can reach 50–80 mM during ischaemia and sustained exposure can trigger death in fetal rat neuronal cultures through enhanced Na^+^ entry into cells [59,60]. Astrocytes may also play a role in energy supply to neurons under compromised conditions via the “lactate shuttle”, the process by which astrocytes convert glycogen to glucose and glucose to lactate (as reviewed in [61]), which can then be transported to neurons and used for oxidative metabolism.

The excessive rise in lactate concentration seen in three animals in the ischaemia-high dose group, despite no significant increase in seizure activity, may be due to impaired glucose trafficking through the astrocytic syncytium from the blood to the neurons. In turn this may result in astrocytes promoting greater glycogenolysis to supply lactate as a fuel for local neuronal function. Alternatively, the decrease in blood PaO_2_ raises the possibility that the combination of seizure activity with greater background neural activity may have exceeded the capacity for oxidative metabolism, with lactate being generated from both neurons and astrocytes by anaerobic metabolism. The secondary increase in impedance, a measure of cytotoxic oedema [32], after brain ischaemia is a result of increased intracellular water uptake, predominantly in the glial cells of the grey matter [32,62,63]. Thus the present data suggest that high-dose infusion was associated with disruption of the astrocytic syncytium, which potentiated intracellular swelling, most likely through impaired local ion homoeostasis.

In conclusion, this study suggests that while low dose infusion of a connexin 43 mimetic peptide that blocks gap junction hemichannel opening may prove to be a clinically useful therapeutic intervention following perinatal ischaemia [13], higher doses may be associated with adverse effects on neural recovery and on survival, speculatively, by uncoupling gap junction communication within the astrocytic syncytium.

## 3. Experimental Section

### 3.1. Fetal Surgery

All procedures were approved by the Animal Ethics Committee of The University of Auckland. In brief, 20 time-mated Romney/Suffolk fetal sheep were instrumented using sterile technique at 118–124 days gestation (term is 145). Food, but not water was withdrawn 18 h before surgery. Ewes were given 5 mL of Streptocin (procaine penicillin (250,000 IU/mL) and dihydrostreptomycin (250 mg/mL, Stockguard Labs Ltd, Hamilton, New Zealand)) intramuscularly for prophylaxis 30 min prior to the start of surgery. Anesthesia was induced by I.V. injection of Alfaxan (Alphaxalone, 3 mg/kg, Jurox, Rutherford, New South Wales, Australia), and general anesthesia maintained using 2–3% isoflurane in O_2_. The depth of anesthesia, maternal heart rate and respiration were constantly monitored by trained anesthetic staff. Ewes received a constant infusion isotonic saline drip (at an infusion rate of approximately 250 mL/h) to maintain fluid balance.

Following a maternal midline abdominal incision and exteriorization of the fetus, both fetal brachial arteries were catheterized with polyvinyl catheters to measure mean arterial blood pressure. An amniotic catheter was secured to the fetal shoulder. ECG electrodes (Cooner Wire Co., Chatsworth, California, USA) were sewn across the fetal chest to record fetal heart rate. The vertebral-occipital anastamoses were ligated and inflatable carotid occluder cuffs were placed around both carotid arteries [4,64]. A 3S Transonic ultrasonic flow probe (Transonic systems, Ithaca, NY) was placed around the right carotid artery. Using a 7 stranded stainless steel wire (AS633-5SSF; Cooner Wire Co.), two pairs of EEG electrodes (AS633-5SSF; Cooner Wire Co.) were placed on the dura over the parasagittal parietal cortex (10 mm and 20 mm anterior to bregma and 10 mm lateral) and secured with cyanoacrylate glue. A reference electrode was sewn over the occiput. A further two electrodes were sewn in the nuchal muscle to record electromyographic activity as a measure of fetal movement and a reference electrode was sewn over the occiput. A thermistor was placed over the parasagittal dura 30 mm anterior to bregma. An intracerebroventricular catheter was placed into the left lateral ventricle (6 mm anterior and 4 mm lateral to bregma). The uterus was then closed and antibiotics (80 mg Gentamicin, Pharmacia and Upjohn, Rydalmere, New South Wales, Australia) were administered into the amniotic sac. The maternal laparotomy skin incision was infiltrated with a local analgesic, 10 mL 0.5% bupivacaine plus adrenaline (AstraZeneca Ltd., Auckland, New Zealand). All fetal catheters and leads were exteriorized through the maternal flank. The maternal long saphenous vein was catheterized to provide access for post-operative maternal care and euthanasia.

### 3.2. Post-Operative Care

Sheep were housed together in separate metabolic cages with access to food and water *ad libitum*. They were kept in a temperature-controlled room (16 ± 1 °C, humidity 50 ± 10%), in a 12 h light/dark cycle. Antibiotics were administered daily for four days I.V. to the ewe (600 mg Benzylpencillin Sodium, Novartis Ltd, Auckland, New Zealand, and 80 mg Gentamicin, Pharmacia and Upjohn). Fetal catheters were maintained patent by continuous infusion of heparinized saline (20 U/mL at 0.15 mL/h) and the maternal catheter maintained by daily flushing.

### 3.3. Data Recording

Data recordings began 24 h before the start of the experiment and continued for the remainder of the experiment. Data were recorded and saved continuously to disk for off-line analysis using custom data acquisition programs (LabView for Windows, National Instruments, Austin, Texas, USA). Arterial blood samples were taken for pre-ductal pH, blood gas, base excess (Ciba-Corning Diagnostics 845 blood gas analyser and co-oximeter, Massachusetts, USA), glucose and lactate measurements (YSI model 2300, Yellow Springs, Ohio, USA). All fetuses had normal biochemical variables for their gestational ages [65,66].

### 3.4. Experimental Protocols

Experiments were performed at 128 ± 1 days gestation. Ischaemia was induced by reversible inflation of the carotid occluder cuffs with saline for 30 min. For Cx43 hemichannel blocking, a peptide (H-Val-Asp-Cys-Phe-Leu-Ser-Arg-Pro-Thr-Glu-Lys-Thr-OH (Auspep, Vic, AU)) that mimics the second extracellular loop of Cx43 (“Peptide 5” reported in [51]) was infused into the lateral ventricle via the intracerebroventricular catheter attached to an external pump. Control fetuses received cerebral ischaemia followed by infusion of the vehicle (ischaemia-vehicle, *n* = 7). The ischaemia-low dose group (*n* = 6) received 50 μmol/kg/h for one h followed by 50 μmol/kg/24 h for 24 h, and the ischaemia-high dose group (*n* = 6) received 50 μmol/kg/h for 25 h dissolved in artificial cerebrospinal fluid (aCSF), at a rate of 1 mL/h for 25 h starting 90 min after the end of the occlusion. Animals were killed at seven days with an overdose of sodium pentobarbitone (9 g I.V. to the ewe; Pentobarb 300, Chemstock International, Christchurch, N.Z.).

### 3.5. Data Analysis

Seizure activity was quantified as number, duration and peak amplitude of seizures between 22–26 h (time of peak EEG power) for each animal in the ischaemia-high dose group. This was correlated with lactate concentration at day one. The time of onset of the first seizure in each animal in both groups was determined. Seizures were defined as previously described by Scher *et al.* [67]. Seizures were visually defined on the raw EEG recording as sudden repetitive, evolving stereotyped waveforms lasting at least 10 seconds with an amplitude greater than 20 μV. Sleep state cycling was identified as alternating periods of high (non-rapid eye movement) and low (rapid eye movement) amplitude activity, with each period lasting approximately 20 min. Data was analyzed using ANOVA or repeated measures ANOVA, followed by the Tukey post-hoc test when a significant difference was found. Statistical significance was accepted when *p* < 0.05.

## 4. Conclusions

After global cerebral ischaemia, low dose mimetic peptide infusion was associated with an enhanced recovery of EEG power seven days after the insult, which was not seen with the high dose infusion. High dose mimetic peptide infusion was associated with a greater secondary increase in impedance, lower blood oxygen tension, and a trend towards a higher lactate concentration as well as greater mortality compared to animals that received vehicle infusion. We speculated that the deleterious effects seen with the high dose infusion were attributable to blockade or uncoupling of connexin 43 gap junctions in addition to hemichannels.

## Figures and Tables

**Table 1:** pH, blood gas, lactate and glucose data before and two, four and six hours after 30 min of global cerebral ischaemia, followed by a daily sample until day seven in the ischaemia-vehicle, -low and -high dose groups.

pH	Baseline	2 h	4 h	6 h	1 day	2 day	7day
**Ischaemiavehicle**	7.40 ± 0.01	7.39 ± 0.01	7.41 ± 0.01	7.40 ± 0.01	7.375 ± 0.02	7.39 ± 0.01	7.36 ± 0.01
**Ischaemia-low**	7.40 ± 0.01	7.40 ± 0.01	7.41 ± 0.01	7.41 ± 0.01	7.38 ± 0.02	7.38 ± 0.01	7.35 ± 0.01 *
**Ischaemia-high**	7.40 ± 0.01	7.40 ± 0.01	7.42 ± 0.01	7.41 ± 0.01	7.31 ± 0.05 †	7.39 ± 0.01	7.35 ± 0.00

**PCO_2_** **(mmHg)**	**Baseline**	**2 h**	**4 h**	**6 h**	**1 day**	**2 day**	**7 day**

**Ischaemiavehicle**	44.6 ± 1.4	42.6 ± 1.6	43.4 ± 1.4	44.9 ± 1.9	44.4 ± 2.7	44.13 ± 1.3	49.4 ± 1.9 †
**Ischaemia-low**	45.1 ± 1.7	44.8 ± 1.2	44.8 ± 1.6	46.6 ± 1.4	44.3 ± 1.4	42.73 ± 1.7	46.2 ± 1.4
**Ischaemia-high**	46.6 ± 0.9	45.2 ± 1.1	45.5 ± 0.7	46.3 ± 0.9	45.3 ± 0.6	47.98 ± 1.6	48.7 ± 2.1

**PO_2_** **(mmHg)**	**Baseline**	**2 h**	**4 h**	**6 h**	**1 day** *	**2 day**	**7 day**

**Ischaemiavehicle**	26.0 ± 1.1	25.8 ± 1.2	25.9 ± 1.25	25.4 ± 1.9	25.0 ± 2.0	24.6 ± 1.5†	23.0 ± 1.1 †
**Ischaemia-low**	23.5 ± 0.9	22.9 ± 0.9	25.2 ± 1.48	24.6 ± 0.5	22.1 ± 1.5	23.4 ± 0.9	22.7 ± 1.0
**Ischaemia-high**	20.7 ± 1.0	21.4 ± 1.0	22.9 ± 1.45	21.2 ± 1.5	18.0 ± 1.2	18.8 ± 2.6	21.6 ± 2.5

**Lactate (mmol/L)**	**Baseline**	**2 h**	**4 h**	**6 h**	**1 day**	**2 day**	**7 day**

**Ischaemiavehicle**	1.1 ± 0.1	2.4 ± 0.5	2.3 ± 0.5	2.0 ± 0.4	4.7 ± 1.1 †	0.94 ± 0.1	1.2 ± 0.1
**Ischaemia-low**	1.1 ± 0.1	2.1 ± 0.3	2.0 ± 0.2	1.6 ± 0.2	2.3 ± 0.5 †	1.46 ± 0.2	1.1 ± 0.0
**Ischaemia-high**	1.2 ± 0.1	2.4 ± 0.3	2.1 ± 0.2	1.9 ± 0.2	10.2 ± 3.3 †	4.21 ± 1.9	1.1 ± 0.1

**Glucose (mmol/L)**	**Baseline**	**2 h**	**4 h**	**6 h**	**1 day**	**2 day**	**7 day**

**Ischaemiavehicle**	0.9 ± 0.1	1.3 ± 0.1 †	1.3 ± 0.1 †	1.3 ± 0.1†	1.5 ± 0.1 †	0.9 ± 0.1	1.0 ± 0.1
**Ischaemia-low**	0.8 ± 0.1	1.3 ± 0.1 †	1.2 ± 0.1 †	1.2 ± 0.1†	1.1 ± 0.1 †	1.0 ± 0.1	0.9 ± 0.1
**Ischaemia-high**	0.8 ± 0.1	1.0 ± 0.1	1.0 ± 0.1	0.9 ± 0.1	1.4 ± 0.2 †	0.9 ± 0.1	0.8 ± 0.2

Data are mean ± SEM,

* *p* < 0.05 ischaemia-vehicle *vs* ischaemia-high dose,

† *p* < 0.05 *vs* baseline. For brevity days 3–6 have been omitted from this table as no significant differences occurred.
